# COMPASS: Navigating the Rules of Scientific Engagement

**DOI:** 10.1371/journal.pbio.1001552

**Published:** 2013-04-30

**Authors:** Brooke Smith, Nancy Baron, Chad English, Heather Galindo, Erica Goldman, Karen McLeod, Meghan Miner, Elizabeth Neeley

**Affiliations:** COMPASS

## Abstract

COMPASS shares a decade of experience in helping scientists become effective leaders by navigating a path from outreach to meaningful engagement with journalists and policymakers.

In an era of heightened competition for scarce research positions and funding, the mantra of modern academia—“publish or perish"—continues to intensify [1]. Scientists are under increasing pressure to produce as many publications as possible in “high-impact" journals to raise their profile among peers and influence their discipline. Yet, in recent years, another measure of significance also has been on the rise—one that focuses on a scientist's reach beyond their field and captures societal impact [2].

More than a decade ago, Jane Lubchenco (a marine ecologist who recently stepped down as Under Secretary of Commerce for Oceans and Atmosphere and Administrator of the US National Oceanic and Atmospheric Administration) codified the idea of a “new social contract for science" [3]. She asserted that society expects two outcomes from its investment of public funds in science: “the production of the best possible science and the production of something useful." Lubchenco challenged scientists to consider not only making their research relevant to today's most pressing problems, but also to embrace their responsibility to share their findings. She urged them to invoke “the full power of the scientific enterprise in communicating existing and new understanding to the public and to policymakers, and in helping society move toward sustainability through a better understanding of the consequences of policy action—or inactions."

As humans continue to push the planet toward a rapid and irreversible state shift [4], this need could not be greater. Yet, finding time to serve society or engage outside of academia can seem impossible. The need to juggle grant writing, teaching, mentoring, and university service, not to mention personal lives, leaves little time for anything else. At a time when policymakers require the expertise of scientists more than ever to solve global challenges, many scientists see the demands to more fully engage with those outside of the ivory tower [5] as just one of many competing priorities.

Knowing it would take more than a call to action, in 1999 Lubchenco and other like-minded colleagues created a nonprofit organization called COMPASS. COMPASS was founded on the premise that ocean scientists, in particular, had a wealth of knowledge that was not reflected in public understanding or policy and management practices. While exciting marine research and new insights were rapidly emerging, those outside the marine science community knew little about them.

COMPASS' mission has been to bridge that gap. Over the past decade, our approach has evolved, reflecting shifts in the culture and practice of science, dramatic changes in the media landscape, and our experience as we pioneer and try new things. Our successes, our failures, and our challenges have taught us that the most effective science communication requires individual and collective commitment to preparation and practice, as well as peer support for reaching outside academia. Scientists need a network of other scientists to encourage and embolden them in their efforts.

Science communication was once considered primarily a unidirectional conveyance of information, based on the assumption that if scientists and other experts could convey their knowledge to the public, typically through “data dumps," society's problems could be solved (i.e., if you knew what I know, you would believe what I believe). This perspective, “the science deficit model of the public", is explored in a body of communications literature [6]–[8]. We know it does not work [9].

Communications is not *only* about speaking in a clear, compelling, and relevant manner, nor simply about promoting findings. Effective communications is an integrated process of understanding your audience and connecting with that audience on their terms. It requires listening as well as talking.

As practitioners within the evolving field of science communication, we've also adapted our approach to one that facilitates dialogue and encourages engagement. We've learned that if scientists want to have impact beyond their disciplines and in the world, communications must be central to their enterprise [10]. This is why academia should reconsider its measures of success and make communication training an integral part of graduate-level education.

## Through Media to the Public and Policymakers

At its inception, COMPASS focused on getting ocean issues onto the social agenda. We began by helping scientists through traditional media outreach, aiming to achieve national and international visibility for fisheries issues, marine protected areas, and ecosystem-level changes. Some of our most visible efforts involved publicizing papers with newsworthy messages such as “90% of the big fish are gone" [11], or international collaborations revealing that the root cause of environmental degradation around the world and in places like the Chesapeake Bay could often be traced back to overfishing [12]. We also helped scientists shed light on important societal issues that led first to industry resistance, then revolution, as when Naylor et al. [13] showed for the first time that aquaculture can be either a gain or a drain on food production, depending on the species farmed.

Much of our work has helped scientists get their research featured in news stories. These busy researchers approached COMPASS for help because they understood that media coverage is a critical, if complex, component of political agenda-setting [14],[15]. Many also learned that news coverage can be related to a significant citation bump in the scientific literature—stories that are covered in high-profile mainstream media, or today through social media, get attention far beyond the reach of the journal in which they were published [16]–[18] (and see Table 1).

**Table 1:** Citations for publications with COMPASS outreach support.

Paper	Citations
Naylor et al. (2000) Effect of aquaculture on world fish supplies. Nature 405: 1017–1024.	795
Watson & Pauly (2001) Systematic distortions in world fisheries catch trends. Nature 414: 534–536.	214
Jackson et al. (2001) Historical overfishing and the recent collapse of coastal ecosystems. Science 293: 629–637.	1,905
Roberts et al. (2001) Effects of marine reserves on adjacent fisheries. Science 294: 1920–1923.	402
Harvell et al. (2002) Climate warming and disease risks for terrestrial and marine biota. Science 296: 2158–2162.	653
Myers & Worm (2003) Rapid worldwide depletion of predatory fish communities. Nature 423: 280–283.	953
Springer et al. (2003) Sequential megafaunal collapse in the North Pacific Ocean: an ongoing legacy of industrial whaling? Proc Natl Acad Sci U S A 100: 12223–12228.	217
Coleman et al. (2004) The impact of United States recreational fisheries on marine fish populations. Science 305: 1958–1960.	138
Worm et al. (2005) Global patterns of predator diversity in the open oceans. Science 309: 1365–1369.	108
Krkosek et al. (2005) Transmission dynamics of parasitic sea lice from farm to wild salmon. Proceedings of the Royal Society B: Biological Sciences 272: 689–696.	101
Worm et al. (2006) Impacts of biodiversity loss on ocean ecosystem services. Science 314: 787–790.	802
Krkosek et al. (2007) Declining wild salmon populations in relation to parasites from farm salmon. Science 318: 1772–1775.	126
Halpern et al. (2008) A global map of human impact on marine ecosystems. Science 319: 948–952.	546
Costello et al. (2008) Can catch shares prevent fisheries collapse? Science 321: 1678–1681.	152
Worm et al. (2009) Rebuilding global fisheries. Science 325: 578–585.	288
Schindler et al. (2010) Population diversity and the portfolio effect in an exploited species. Nature 465: 609–612.	92

List of the publications whose authors worked with COMPASS to promote their research findings. Citation count from ISI Web of Science as of February 10, 2013.

When working on outreach efforts, we make a verbal contract with the authors. It's up to them, with our help, to determine *their* key messages—what it is they want to communicate and to whom? They must agree to take the necessary time to prepare and to commit to making themselves available to the journalists, and later policymakers, who will want to talk to them.

We remind the authors that making a splash in the mainstream press tends to incite controversy, whether over the science itself, the communication of it, or both. Backlash is never pleasant, but it is not necessarily negative [5]. In our experience, when the science is robust, and authors are committed to the questions instead of the results, criticism can catalyze productive collaborations and push the field forward. The paper “Impacts of Biodiversity Loss on Ocean Ecosystem Services" [19], which became known as “the end of seafood by 2048", was initially met with outrage from some traditional fisheries scientists. Ultimately, however, this led to collaboration [20]. Twenty-two leading scientists and dozens of graduate students from opposing world views (marine ecology and traditional fisheries) formed a working group at the National Center of Ecological Analysis and Synthesis (NCEAS) and compiled new datasets to reach a consensus view of the state of world fisheries [21]. Of course, efforts to further refine understanding of fisheries continue [22].

## From Sharing to Engaging

Sharing scientific findings broadly is what allows them to be visible and can bring together a wide range of stakeholders and decision makers around these issues. The real work is not only in broadcasting the results, but also in connecting the authors with those who can advance the conversation—whether that means new collaborations, developing regulatory policy, or taking action.

Effective communication invites engagement. Scientists who enter into social dialogues bring much more than just the results of their research. They bring key insights into *how* we understand the systems they study. This perspective can particularly enrich public policy discussions. For example, does what we've learned from studying no-take marine reserves in one place apply to another? If so, why, and under what circumstances? Scientists can speak to potential risks, uncertainties, tradeoffs and the implications of science for a particular decision. In many cases, scientists are able to help clarify the choices before a policymaker by recasting the questions they ask and providing a different framework for approaching the challenge at hand. For example, perhaps the tradeoff is not as simple as habitat conservation versus the economic value of the fishing industry, but instead involves more complex tradeoffs among a broader range of societal benefits that flow from our coastal waters.

Effective science-policy dialogue hinges on having the right people in the room at the right time, and who are fully committed to the process. It requires keen insight into what is on a policymaker's desk and the complex issues at play. Beyond formal advisory panels, scientists often don't know where or how to look for opportunities to get involved. The effort to get started can be overwhelming. COMPASS helps by tracking the decision-making landscape—legislatures, agencies, and others—to identify opportunities where scientific insights can advance the policy dialogue. We broker carefully timed connections between key policymakers and scientists and support scientists to navigate what is often a very foreign environment.

One such opportunity arose in 2004, when two high-level commissions recommended ecosystem-based management (EBM) as the cornerstone of a new vision for ocean policy. Ensuing policy discussions of EBM did not fully reflect the underlying science. To bridge this gap, we engaged leading marine scientists to develop a scientific consensus statement about marine ecosystems for policymakers. We provided insights into the complex policy context and supported the scientists to synthesize the science. The language in this statement, signed by over 200 scientists, now appears in multiple agency and legislative contexts, including the implementation plan for the National Ocean Policy.

Some of these scientists engaged directly with policymakers to share insights from the consensus statement. These interactions sparked opportunities to further advance the science, including new research initiatives focused on how to measure ecosystem services [23], science to inform tradeoffs [24],[25], new measures of ocean health [26],[27], and syntheses of EBM science and practice [28]–[30]. Importantly, all of this work was the result of many dialogues between scientists and policymakers to make sure that the science would be relevant and useful.

**Figure 1:**
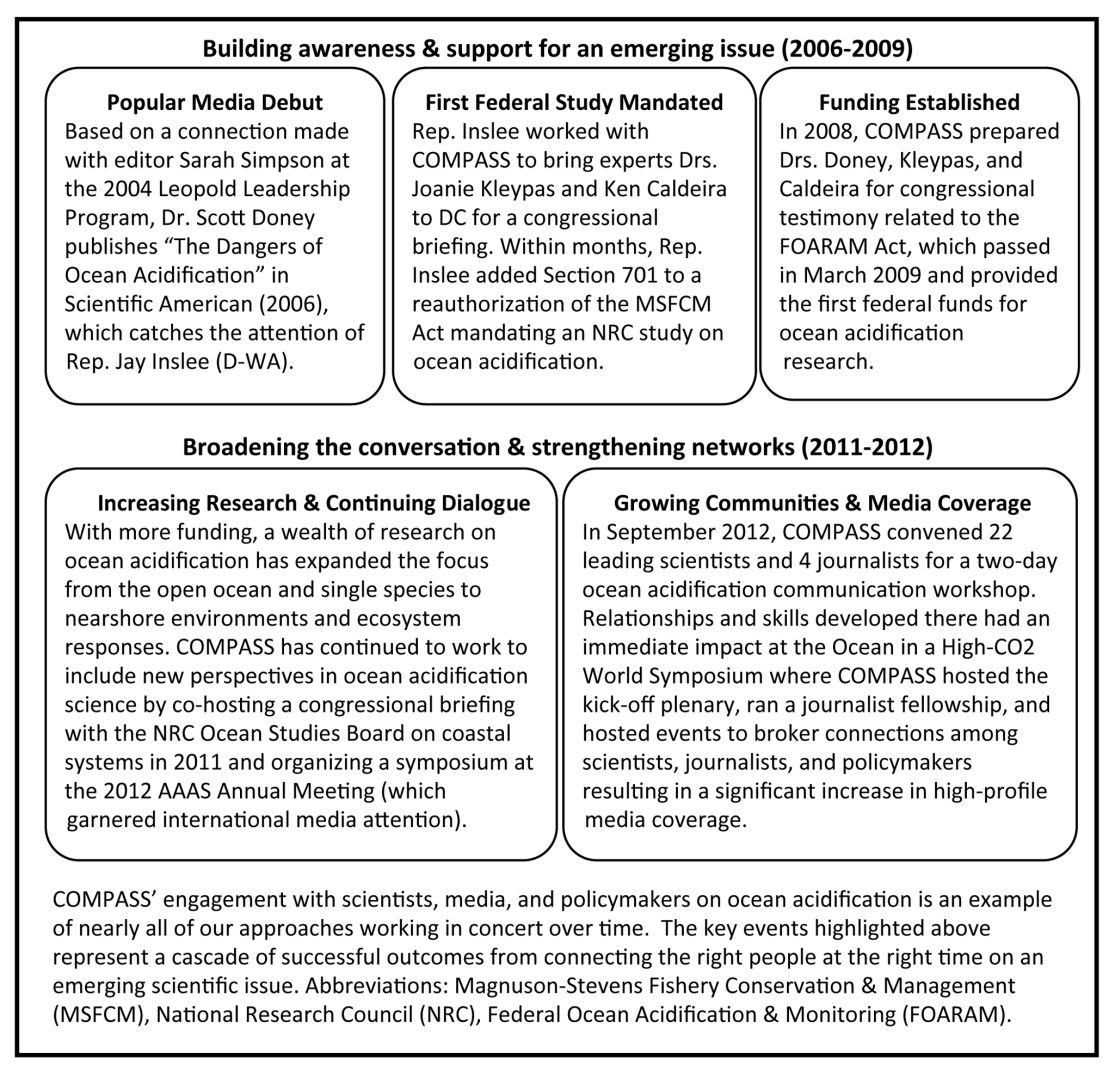
Science engagement and ocean acidification policy.

Science and policy interactions can take years to play out (Figure 1). In 2004 we helped some of the first scientists working on ocean acidification to connect with key journalists, which resulted in high-profile stories that attracted the attention of both the public and policymakers. We then brokered connections with policymakers to give them direct access to the scientists. The result of these and others' efforts has been greater public awareness of the issue of ocean acidification, more research dollars available, and some new policies and practices based on the growing body of scientific insights and evidence.

## Towards Culture Change

We know, however, that practical considerations can dissuade even the most willing participants from engaging. When it comes to science outreach, researchers cite not only a lack of time and funding, but also the lack of knowledge and training as an impediment [31]. The appetite for this training and the support from within varies among institutions. There can be serious barriers to engagement, even disincentives. Cultural bias against engagement afflicts some universities, departments, and disciplines, which not only fail to reward such efforts but actively discourage them.

This is why a network of support is so important. In the past 10 years, COMPASS has created and led communication trainings for not only the Leopold Leadership Fellows, but also for hundreds of faculty, researchers, and graduate students in the United States and beyond. We design vertically integrated workshops that include both senior as well as promising young scientists to instigate a support network that transcends hierarchy. Some workshops, in addition to helping participants become more effective communicators, bring government, nongovernmental organization, and academic scientists together to seed new collaborations, inspire cross-pollination, and help entire scientific communities to more effectively engage with society. Our cooperative learning approaches are designed to help scientists build networks of support that carry on long after the workshop. Last year's training of scientists from Scandinavian countries working on low oxygen zones in the Baltic Sea has led to the creation of the Vega Fellows in Communication and Leadership, which is based on the Leopold Leadership program.

Before each workshop, we survey participants about their hopes, fears, and challenges in connecting to the media. Consistent with previous findings [32], we see a widespread fear of being misquoted, and discomfort with the lack of control over interviews and stories in general. Scientists are nervous about how their peers will react to their engagement with the press. Yet, participants say they are willing to engage because they hope that sharing their knowledge with the broader world will make a difference.

COMPASS goes beyond what scientists may think of as “media training." Our interactive workshops are shaped around the intrinsic link between communication and leadership—they are about engagement [10]. Scientists who can clearly explain a research finding and why it matters are poised to succeed not just in outreach, but also in grant writing, interdisciplinary collaborations, teaching, and other essential roles. Being a good communicator is not a tradeoff; it is a key component of scientific success. Like most other elements of a strong academic career, it's a skill that may be rooted in natural talent and personal interest, but can always be further developed by training, preparation, and practice.

Across a wide swath of disciplines, there are increasing demands for better training to develop science communication and knowledge brokering skills [33]–[36]. If communication is to become an authentic component of professional competency, it must be systematically integrated into the values, identities, and systems for justifying decisions within scientific communities [37] (and references therein). Academic institutions and tenure committees must measure and reward time and effort devoted to outreach. And that, we're keenly aware, will require dedicated leadership and collective effort to change the culture of science.

## Conclusion

Our work at the intersection of science, policy, and media to support science and scientists is constantly evolving. Our approach—empowering individual scientists, connecting communities, and creating opportunities for transformative conversations—has helped scientists traverse traditional disciplinary boundaries and effectively address pressing environmental issues. Inspired by them, and in response to requests from the broader community, we have expanded beyond ocean science to support a broader swath of scientists.

COMPASS is dedicated to helping scientists become more effective communicators as well as creating enthusiasm, appetite, and a conviction in its importance. While the number of newspaper headlines, blog posts, or congressional briefings a scientist is connected to may not always be lauded in academia, the journey to get there is often rewarding.

Through our work, we've helped develop an ever-expanding network of scientist communicators and leaders who support each other's engagement inside and outside of academia, and are championing a reward structure for these efforts. Increasingly, scientists who endeavor to bring science closer to society will find themselves in good company.

## Abbreviations

EBM: ecosystem-based management
